# Short-read full-length 16S rRNA amplicon sequencing for characterisation of the respiratory bacteriome of captive and free-ranging African elephants (*Loxodonta africana*)

**DOI:** 10.1038/s41598-024-65841-4

**Published:** 2024-06-26

**Authors:** Lauren C. Martin, Michaela A. O’Hare, Giovanni Ghielmetti, David Twesigomwe, Tanya J. Kerr, Rachiel Gumbo, Peter E. Buss, Natasha Kitchin, Sian M. J. Hemmings, Michele A. Miller, Wynand J. Goosen

**Affiliations:** 1 Department of Psychiatry, Faculty of Medicine and Health Sciences, Stellenbosch University, PO Box 241, Cape Town, 8000 South Africa; 2 South African Medical Research Council/Stellenbosch University Genomics of Brain Disorders Unit, Cape Town, South Africa; 3 Division of Molecular Biology and Human Genetics, Faculty of Medicine and Health Sciences, Stellenbosch University, PO Box 241, Cape Town, 8000 South Africa; 4 South African Medical Research Council Centre for Tuberculosis Research, Division of Molecular Biology and Human Genetics, Faculty of Medicine and Health Sciences, Stellenbosch University, PO Box 241, Cape Town, 8000 South Africa; 5 Section of Veterinary Bacteriology, Institute for Food Safety and Hygiene, Vetsuisse Faculty, University of Zurich, Winterthurerstrasse 270, 8057 Zurich, Switzerland; 6 Sydney Brenner Institute for Molecular Bioscience, Faculty of Health Sciences, University of the Witwatersrand, Johannesburg, South Africa; 7 Division of Human Genetics, National Health Laboratory Service, and School of Pathology, Faculty of Health Sciences, University of the Witwatersrand, Johannesburg, South Africa; 8 South African National Parks, Veterinary Wildlife Services, Kruger National Park, Skukuza, South Africa

**Keywords:** 16S rRNA sequencing, Respiratory microbiome, Elephant microbiome, iSeq 100, Translational research, Infection, Next-generation sequencing, Targeted resequencing

## Abstract

Hypervariable region sequencing of the 16S ribosomal RNA (rRNA) gene plays a critical role in microbial ecology by offering insights into bacterial communities within specific niches. While providing valuable genus-level information, its reliance on data from targeted genetic regions limits its overall utility. Recent advances in sequencing technologies have enabled characterisation of the full-length 16S rRNA gene, enhancing species-level classification. Although current short-read platforms are cost-effective and precise, they lack full-length 16S rRNA amplicon sequencing capability. This study aimed to evaluate the feasibility of a modified 150 bp paired-end full-length 16S rRNA amplicon short-read sequencing technique on the Illumina iSeq 100 and 16S rRNA amplicon assembly workflow by utilising a standard mock microbial community and subsequently performing exploratory characterisation of captive (zoo) and free-ranging African elephant (*Loxodonta africana*) respiratory microbiota. Our findings demonstrate that, despite generating assembled amplicons averaging 869 bp in length, this sequencing technique provides taxonomic assignments consistent with the theoretical composition of the mock community and respiratory microbiota of other mammals. Tentative bacterial signatures, potentially representing distinct respiratory tract compartments (trunk and lower respiratory tract) were visually identified, necessitating further investigation to gain deeper insights into their implication for elephant physiology and health.

## Introduction

African savanna elephants (*Loxodonta africana*) are an endangered species^1^, mainly due to poaching and habitat loss associated with human activities^2^. To date, approximately 415,000 African elephants are estimated to roam the African continent, of which 44,326 are reported to reside within the geographical boundaries of South Africa^1,3^. The Kruger National Park is home to the largest population of wild African elephants, with an estimated count of 31,200 individual animals^3^. In addition, there are approximately 126 captive or managed African elephants reported in South Africa^4^. These elephant populations face further threats from infectious pathogens, such as elephant endotheliotropic herpesvirus (EEHV)^5^ and tuberculosis (TB)^6–8^. The presence of disease in these populations can lead to detrimental consequences for the maintenance of a stable ecological niche^9^. These concerns highlight the need for tools that can easily and cost-effectively monitor the health of these animals to support conservation and ecosystem health.

The microbiome, which describes the distinct microbial communities that occupy the habitable compartments and niches of the host^10^, has been increasingly explored in health-related wildlife conservation efforts. Research has demonstrated that animal immune, gastrointestinal and reproductive health are intrinsically linked to the composition and corresponding functional processes of host-associated microbial communities^11^. In the context of elephant microbiome research, focus has been placed on the characterisation of African and Asian elephant intestinal microbiota in an effort to improve the health status and welfare of these animals^12–19^, restore the microbial diversity of captive elephants^12,14,15,17,19^, and enhance their diet^13,16,20^. Investigation into the respiratory microbiota may enable broad characterisation of the microbiota of healthy elephants, documentation of undescribed microbial diversity, and identification of relationships between microbiota and host disease phenotypes. Furthermore, such exploration may offer valuable insights into the broader respiratory microbial landscape of other mammals, reported as being dominated by Proteobacteria, Actinobacteriota, Firmicutes, and Bacteroidota phyla^21^, and may eventually aid in mapping pathogen transmission at human-animal interfaces.

The 16S ribosomal RNA (rRNA) gene is ubiquitous across bacterial species and is thus often used as a genetic marker for bacterial and archaeal taxonomic differentiation^22^. Within this gene are nine hypervariable regions, which, when sequenced in isolation or coupled with one or two other regions, provide a representative estimate of bacterial abundance at the genus level. However, it falls short of achieving adequate species-level taxonomic resolution, primarily due to the limited genetic information offered by the selected hypervariable regions^23–25^. Recent evidence has shown that investigation of full-length 16S rRNA gene sequences improves species-level taxonomic classification and minimises the taxonomic bias introduced by targeting specific hypervariable regions^23–25^. To overcome the limitations of short-read sequencing, innovative methods have been applied to reconstruct full-length, or near full-length, bacterial 16S rRNA progenitor molecules, involving full-length amplicon shearing and dual-adapter amplicon ligation^26^, termini tagging^27^, or random intramolecular identifier insertion^28^. Although these approaches yield high-quality assemblies with low estimated error rates, they remain expensive and inaccessible in resource-poor settings^27,28^.

Illumina sequencing platforms have been widely adopted for short-read sequencing in microbiome research^23^. Among these platforms, the Illumina iSeq 100 benchtop instrument represents a compact and cost-effective alternative for sequencing small genomes and amplicons using a streamlined workflow requiring minimal expertise and hands-on time^29,30^. To date, only 300 cycle kits, generating maximum read lengths of 2 × 150 bp, are available for use on this instrument^29^. In targeted metagenomic sequencing applications, bacterial community characterisation on this platform has been limited to the V4 region (~ 254 bp) of the 16S rRNA gene because of the restrictive read lengths. This has also reduced the platform’s ability to sequence larger regions of interest, such as the full-length 16S rRNA gene, which holds greater metataxonomic potential.

**Figure 1:**
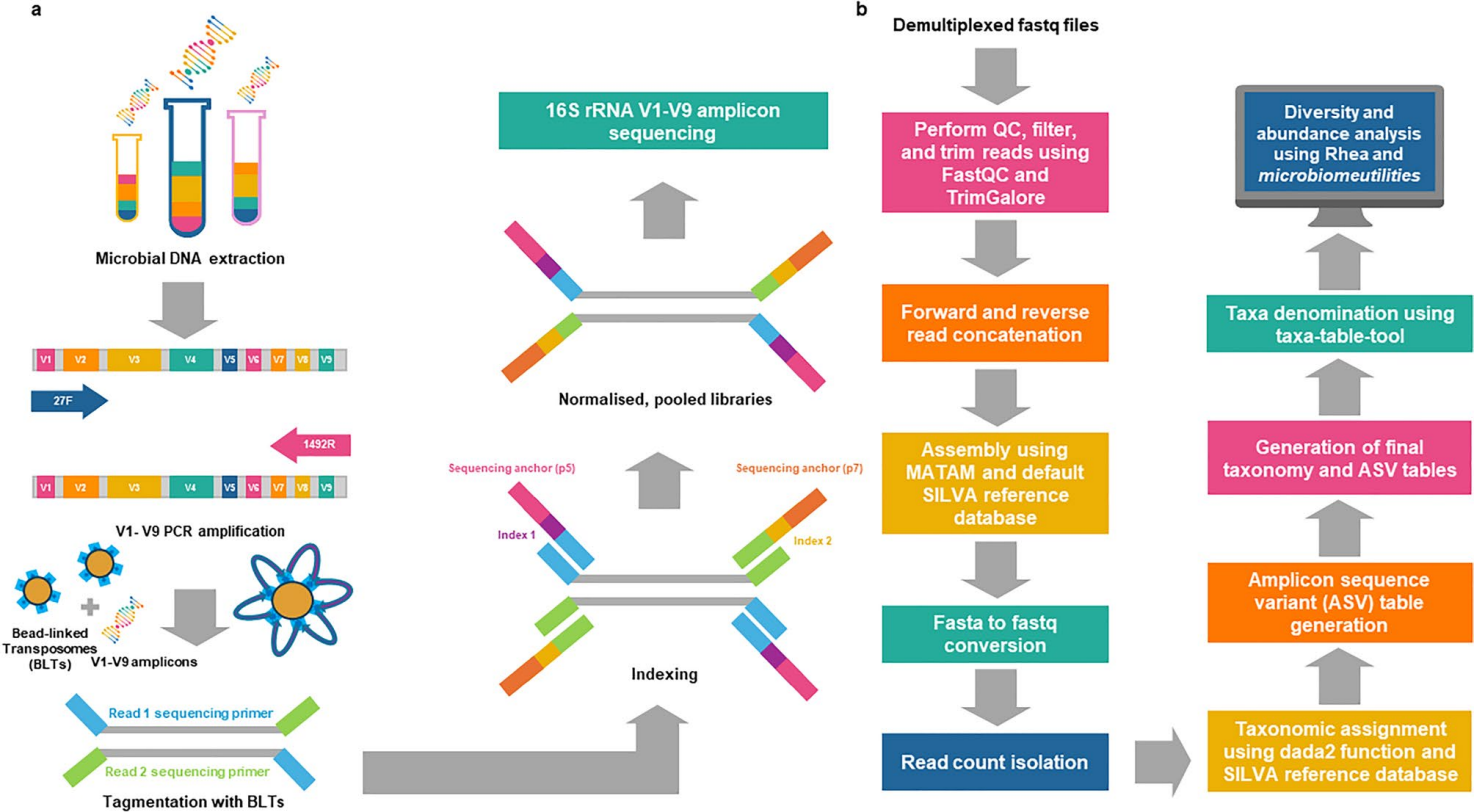
Overview of the library preparation, sequencing, and 16S rRNA amplicon re-assembly. Samples are subject to (**a**) DNA extraction, library preparation, and 150 bp paired-end short-read sequencing on the Illumina iSeq 100, before (**b**) amplicon reconstruction using the 16S-amplicon-seq workflow and microbiome diversity and composition analysis.

To capitalise on the platform’s benefits and address the instrument’s challenges with microbial community characterisation using larger genetic regions of interest, we developed a modified 150 bp paired-end short-read 16S rRNA amplicon sequencing technique and assembly pipeline tailored for the iSeq 100 platform (Fig. 1). The method involves the tagmentation and indexing of full-length 16S rRNA amplicons to ensure compatibility with the 2 × 150 bp read configuration of the instrument, and the subsequent redirection of the generated reads into a bioinformatic gene assembly workflow for reconstruction of the progenitor amplicon, following the sequencing of the prepared libraries.

Therefore, the aim of this study was to assess the feasibility of a modified 150 bp paired-end short-read 16S rRNA amplicon sequencing technique on the Illumina iSeq 100 platform and a bioinformatic gene assembly workflow using a commercial microbial community standard, and respiratory samples (bronchioalveolar lavage (BALF), endotracheal tube (ETT), and trunk wash (TW) fluid) collected from captive (zoo) and free-ranging African elephants. It was anticipated that short-read sequencing of the elephant respiratory samples would yield high-quality near full-length to full-length gene assemblies that, once taxonomically classified, would provide an initial indication of the bacterial communities present in the elephant respiratory system. These results provide an important foundation for advancing knowledge of the respiratory microbiome of African elephants and its contribution to their health.

## Results

Following library preparation and sequencing of the tagmented 16S rRNA amplicon libraries on the iSeq 100 (Fig. 1a), 1.66 Gb of indexed reads were generated. A total of 68.90 ± 0.75% reads passed the sequencing chastity filter (%PF) and 88.58% had a Q-score of ≥ 30. Across the samples, the number of raw reads ranged from 157,010 to 350,656 reads, with an mean count of 228,770.83 reads (SD = 52,632.71). Reads were re-assembled as per the workflow detailed in Fig. 1b. The assembled sequences ranged between 500 and 1696 bp (mean = 868.90; SD = 314.16) and comprised 72,274 to 164,302 sequences, respectively, (mean = 104,779.33; SD = 24,967.16).

### Concordance with the theoretical composition of the microbial standard

**Figure 2:**
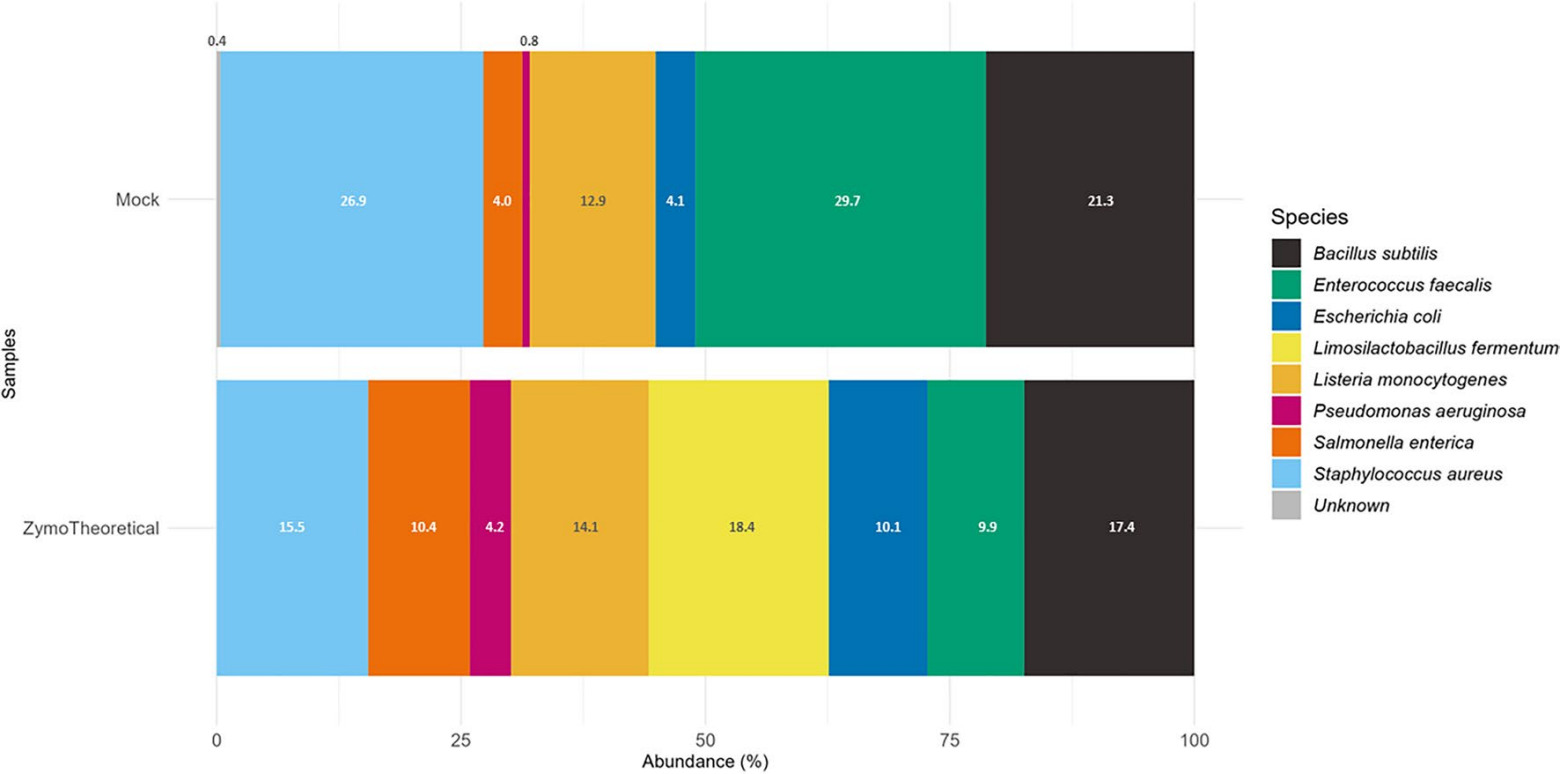
Representation of the concordance between the theoretical composition of the commercial microbial standard and microbial standard sequenced and assembled using the adapted 150 bp paired-end full-length 16S rRNA gene sequencing technique and 16S-amplicon-seq workflow. Horizontal stacked bar charts depicting the composition of sequenced microbial standard and theoretical composition of ZymoBIOMICS™ Microbial Community Standard. A very weak concordance (ρ = 0.117) between the commercial standard and the sequenced mock community was found.

The sequenced commercial microbial community displayed positive, but very weak (ρ = 0.117), concordance with the expected taxa and their abundances (Fig. 2). Seven of the eight expected bacterial species were detected in the sequenced mock microbial community, albeit in abundances differing from the theoretical composition (Fig. 2). Inspection of individual taxon abundances revealed the absence of *Lactobacillus fermentum* (reclassified as *Limosilactobacillus fermentum*) and lower relative abundances of *Escherichia coli* (4.1%), *Pseudomonas aeruginosa* (0.8%) and *Salmonella enterica* (4.0%) relative to the expected composition of the microbial community standard (10.1%, 4.2% and 10.4%, respectively) (Fig. 2). In contrast, *Enterococcus faecalis* (29.7%) and *Staphylococcus aureus* (26.9%) were present in greater abundances in the sequenced mock microbial community relative to the theoretical composition (9.9% and 15.5%, respectively) (Fig. 2).

### BALF samples and free-ranging elephant samples display greater bacterial diversity

**Figure 3:**
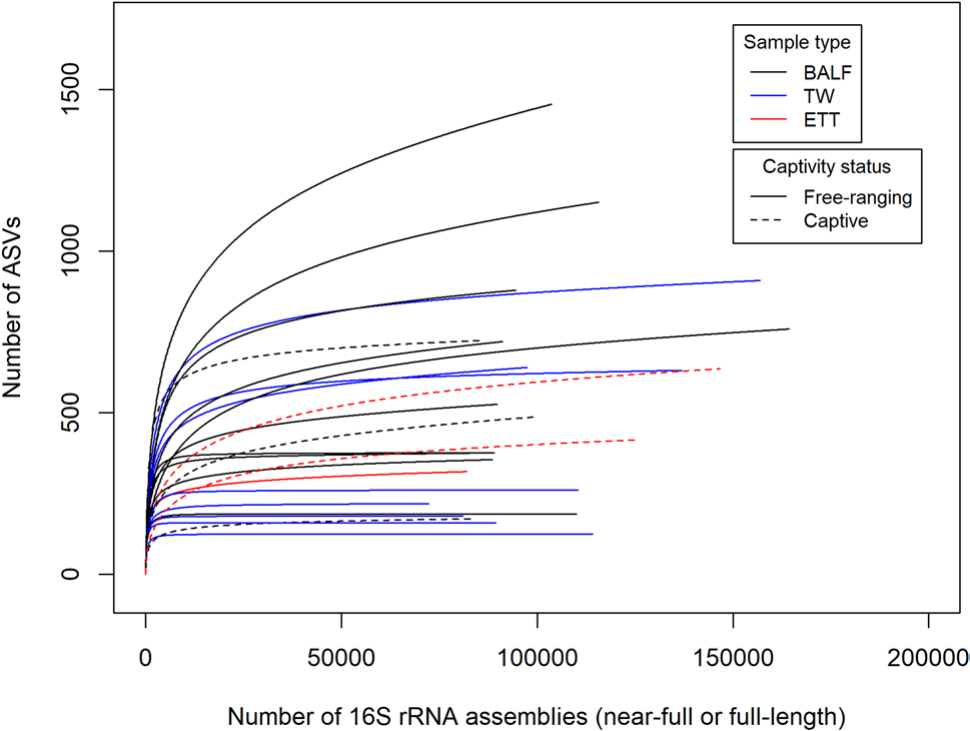
Rarefaction curves of elephant respiratory samples delineated according to sample type and captivity status. The horizontal axis shows the total number of assembled reads. The vertical axis depicts the number of ASVs observed. The majority of the samples (22/24) achieved a plateau, indicating that diversity was sufficiently captured. *BALF* bronchoalveolar lavage fluid, *ETT* endotracheal tube wash, *TW* trunk wash.

A total of 12,660 amplicon sequence variants (ASVs) were identified in the dataset. The average number of ASVs across the dataset was 527.50 (SD = 338.81), with 125 and 1454 ASVs representing the minimum and maximum number of ASVs present in individual samples, respectively (Fig. 3). Rarefaction curves for the majority (22/24) of the samples reached a clear plateau, indicating that diversity was, for most samples, sufficiently captured (Fig. 3). The BALF samples had the highest average ASV abundance relative to ETT and TW samples (mean BALF = 628.10, SD BALF = 375.02; mean ETT = 456.70, SD ETT = 162.85; mean TW = 390.60, SD TW = 293.51, respectively). Respiratory samples collected from captive zoo elephants (mean = 486.80; SD = 213.40) had a lower mean ASV abundance than those obtained from free-ranging elephants (mean = 538.20, SD = 368.75).

Similar median Shannon index estimates were obtained for BALF (4.42), ETT (4.41), and TW (4.42) samples. However, when examining the Shannon effective numbers, BALF samples exhibited a substantially higher estimate of represented species (121.10) relative to ETT (82.39) and TW (82.86) samples (Table 1). Similarly, the median Simpson index estimates revealed lower diversity in ETT (0.020) and TW (0.020) samples compared to the BALF (0.016) samples. This was also reflected in the Simpson effective number, where the median number of species represented was notably higher in the BALF samples (64) compared to the ETT (51) and TW (51) samples (Table 1).

**Table 1:** Shannon, Shannon effective number, Simpson and Simpson effective number alpha diversity estimates for sample types and captivity status groups.

Sample type					
Alpha diversity estimate	Category	Min	Median	Max	IQR
Shannon index	BALF	3.81	4.80	5.81	0.60
	ETT	3.89	4.41	4.68	0.40
	TW	3.92	4.42	5.40	0.70
Shannon effective number	BALF	45	121	334	66
	ETT	49	82	108	30
	TW	50	83	222	83
Simpson index	BALF	0.01	0.02	0.04	0.01
	ETT	0.02	0.02	0.03	0.01
	TW	0.01	0.02	0.03	0.01
Simpson effective number	BALF	27	64	209	38
	ETT	32	51	62	15
	TW	32	51	107	26
Captivity status					
Shannon index	National Park	3.92	4.68	5.77	0.63
	Zoo	3.81	4.28	5.81	0.52
Shannon effective number	National Park	50	108	319	72
	Zoo	45	72	334	33
Simpson index	National Park	0.01	0.02	0.04	0.01
	Zoo	0.004	0.03	0.03	0.01
Simpson effective number	National Park	27	43	169	33
	Zoo	32	32	209	19

*BALF* bronchoalveolar lavage fluid, *ETT* endotracheal tube wash, *TW* trunk wash, *IQR* inter-quartile range.

Free-ranging elephant respiratory samples had higher median Shannon index (4.68) and Shannon effective number estimates (108) compared to samples collected from captive zoo elephants (Shannon index = 4.28, Shannon effective number = 72) (Table 1). The median Simpson index estimates revealed greater diversity in samples obtained from free-ranging elephants (0.02) compared to captive zoo elephant (0.03) samples. Similar results were reflected in the median Simpson effective number estimates (Table 1).

### Beta diversity

**Figure 4:**
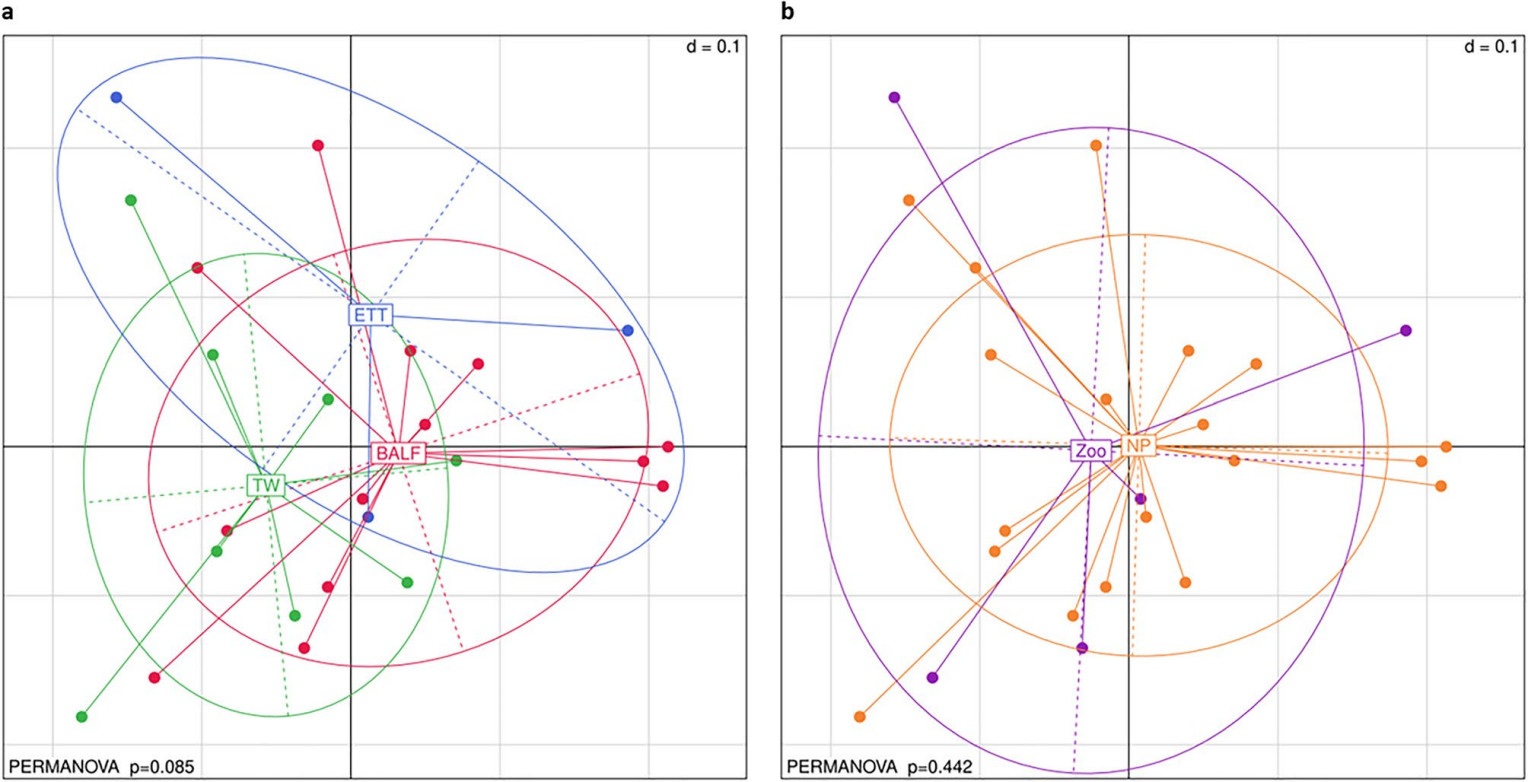
Multidimensional scaling (MDS) plot of generalized Unifrac dissimilarities derived from the bacterial communities of BALF, ETT, and TW respiratory samples of captive (zoo) and free-ranging elephants. Plots are segregated by (**a**) sample type and (**b**) captivity status. Intersample variation between sample type groups trended toward significance (p = 0.085, PERMANOVA). Pairwise comparison revealed near-significant differences in the beta diversity of ETT and TW (*p* = 0.029, *p*_*adj*_ = 0.087). There were no significant differences between free-ranging (National Park) and captive (Zoo) elephants (*p* = 0.442, PERMANOVA). *BALF* bronchoalveolar lavage fluid, *ETT* endotracheal tube wash, *TW* trunk wash, *NP* National Park.

Beta diversity analyses revealed a trend towards significance for differences in diversity between the BALF, ETT and TW samples (*p* = 0.085, PERMANOVA) (Fig. 4a). Subsequent pairwise analyses indicated that significant differences exist between ETT and TW samples (*p* = 0.029, Mann–Whitney Test) before Benjamini–Hochberg correction (*p*_adj_ = 0.087), while no significant differences between the other sample type pairs were identified (Supplementary Fig. S1). No noticeable or statistically significant patterns of dispersion or clustering were identified between captive and free-ranging elephants (*p* = 0.442, PERMANOVA) (Fig. 4b).

### Elephant respiratory samples composed of four main bacterial phyla

The proportions of bacterial taxa varied across the samples. However, of the 15 bacterial phyla detected, only four (Proteobacteria, Actinobacteriota, Firmicutes, and Bacteroidota) displayed relative abundances exceeding 0.1%, collectively representing 98.5% of the total relative abundance across the samples (Table 2, Supplementary Material).

**Table 2:** Relative abundance (%) of prominent phyla in the bacterial profiles of the respiratory samples stratified by sample type and captivity status.

Phylum	Sample type			Captivity status	
	BALF	ETT	TW	National Park	Zoo
Proteobacteria	53.0 ± 36.7	74.5 ± 23.4	33.0 ± 15.8	51.1 ± 30.8	41.3 ± 37.9
Actinobacteriota	9.5 ± 19.2	8.4 ± 13.8	41.8 ± 36.6	22.7 ± 31.3	10.4 ± 19.9
Firmicutes	26.0 ± 25.1	15.9 ± 26.8	2.8 ± 2.3	9.7 ± 13.6	44.9 ± 29.7
Bacteroidota	9.1 ± 14.3	1.2 ± 1.9	21.7 ± 23.4	14.7 ± 19.4	3.4 ± 7.4

*BALF* bronchoalveolar lavage fluid, *ETT* endotracheal tube wash, *TW* trunk wash.

### Potential signature specific to TW and BALF samples visually identified in free-ranging elephants

Within BALF samples, *Pseudomonas*, *Streptococcus* and *Stenotrophomonas* were the top three most abundant genera (21.7%, 8.1%, and 7.6%, respectively) (Supplementary Figs. S2 and S3, Supplementary Material). The TW samples were dominated by *Rothia* (35.9%), with *Pseudomonas* (9.1%) and *Pedobacter* (8.3%) being the second and third most prevalent genera, respectively (Supplementary Figs. S2 and S3, Supplementary Material). The ETT samples showed similar abundances to that of BALF samples, with *Ralstonia*, *Stenotrophomonas* and *Streptococcus* as the top three genera (21.9%, 16.8%, and 15.1%, respectively) (Supplementary Figs. S2 and S3, Supplementary Material).

The top three most abundant genera in respiratory samples of free-ranging elephants were *Pseudomonas*, *Rothia* and *Streptococcus* (19.0%, 15.3%, and 4.8%, respectively), while captive zoo elephants were dominated by *Bacillus*, *Ralstonia* and *Stenotrophomonas* (16.5%, 14.1%, and 13.0%, respectively) (Supplementary Figs. S2 and S3, Supplementary Material). The relative abundances of the top 25 bacterial genera grouped by sample type and ordered by Bray–Curtis distance showed *Rothia*, *Enhydrobacter*, *Moraxella*, and *Chryseobacterium* genera in five of the eight TW samples (Supplementary Fig. S3). *Pseudomonas* formed a significant proportion of five of the ten BALF samples collected from free-ranging elephants (Supplementary Fig. S3).

### Potential genus-level TW visually identified signature persists at species level

**Figure 5:**
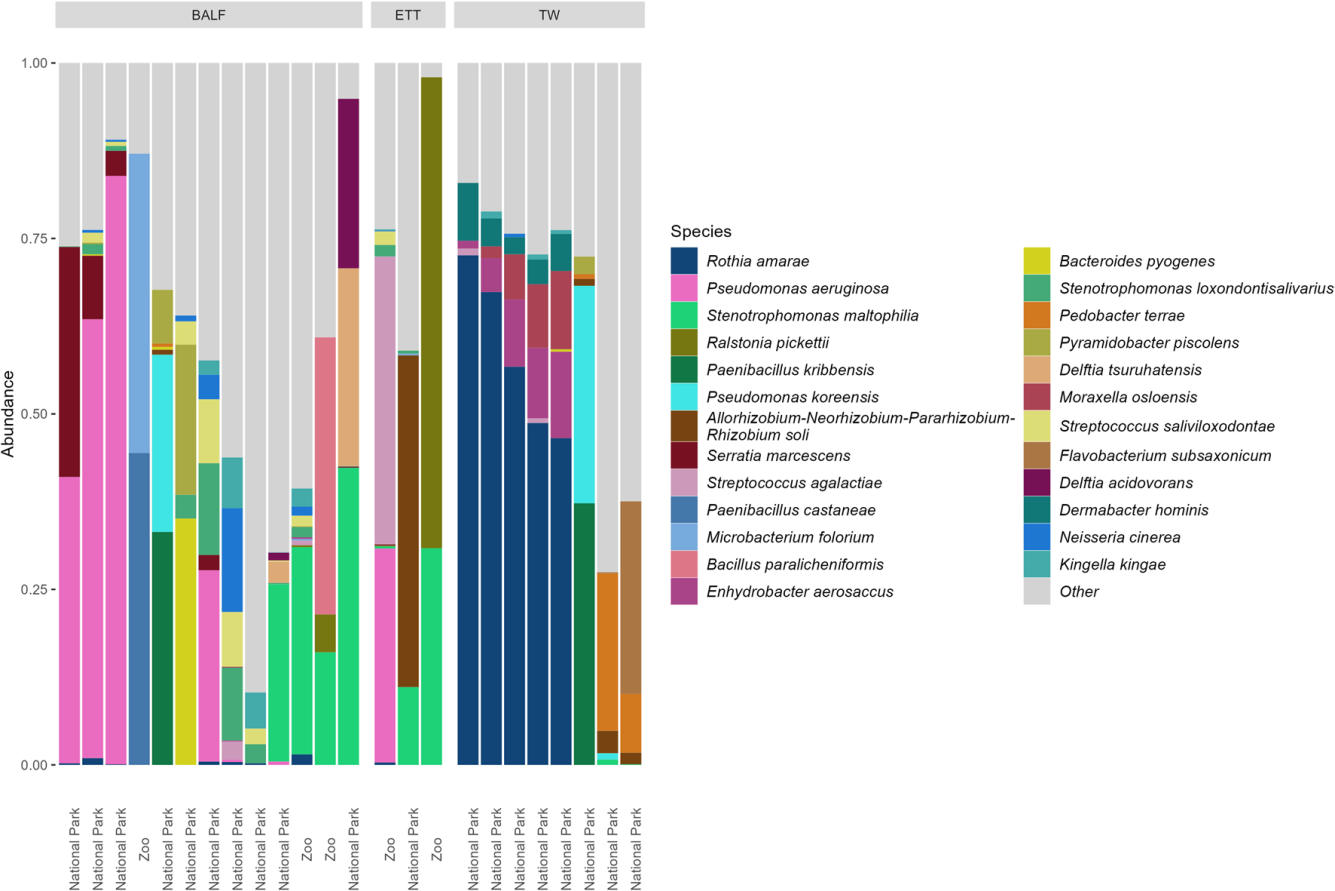
Top 25 most abundant bacterial species present in BALF, ETT, and TW samples collected from captive (zoo) and free-ranging elephants. Relative abundances of these species are displayed with stacked bar charts stratified by sample type. Samples are ordered according to Bray–Curtis dissimilarity. *BALF* bronchoalveolar lavage fluid, *ETT* endotracheal tube wash, *TW* trunk wash.

Within the BALF samples, the most abundant bacterial species observed were *Pseudomonas aeruginosa* (16.6%), *Stenotrophomonas maltophilia* (8.7%), *Serratia marcescens* (3.7%), *Paenibacillus castaneae* (3.4%), and *Microbacterium foliorum* (3.3%) (Fig. 5, Supplementary Fig. S4, Supplementary Material). The ETT samples predominantly consisted of *Ralstonia pickettii* (22.4%), *Allorhizobium-Neorhizobium-Pararhizobium-Rhizobium soli* (15.8%), *Stenotrophomonas maltophilia* (14.1%), *Streptococcus agalactiae* (13.7%), and *Pseudomonas aeruginosa* (10.2%) species (Fig. 5, Fig. 6). *Rothia amarae*, *Enhydrobacter aerosaccus*, *Pseudomonas kribbensis*, *Pseudomonas koreensis*, and *Pedobacter terrae* were among the most abundant species present in the TW samples at abundances of 36.5%, 4.7%, 4.7%, 4.0%, and 4.0% respectively (Fig. 5, Supplementary Fig. S4, Supplementary Material).

The most abundant species within free-ranging elephants were *Rothia amarae* (15.5%), *Pseudomonas aeruginosa* (11.3%), *Stenotrophomonas maltophilia* (4.2%), *Pseudomonas kribbensis* (3.7%), and *Pseudomonas koreensis* (3.0%), while captive zoo elephants were dominated by *Stenotrophomonas maltophilia* (15.4%), *Ralstonia pickettii* (14.6%), *Paenibacillus castaneae* (8.9%), *Microbacterium foliorum* (8.6%), and *Streptococcus agalactiae* (8.3%) (Fig. 5, Supplementary Fig. S4, Supplementary Material).

Visual inspection of the relative abundance of individual samples revealed a compositional pattern of *Rothia amarae*, *Enhydrobacter aerosaccus*, *Moraxella osloensis*, and *Dermabacter hominis* species similar to that of the one identified in the five TW samples at genus level (Fig. 5).

## Discussion

In this study, we adapted an existing hypervariable sequencing technique to explore the feasibility of sequencing the full-length 16S rRNA gene on the Illumina iSeq 100. A Nextflow workflow was developed to streamline gene assembly and enable comprehensive characterisation of the elephant respiratory microbiome.

The application of the sequencing technique and assembly workflow to the mock microbial community and elephant respiratory samples resulted in the retrieval of high quality near full-length to full-length reads. In line with Illumina iSeq 100 i1 v2 reagent kit performance measures^29^, 88.58% of the base calls had a Q-score ≥ 30. Reads were expected to be approximately 1465 bp in length^24^; however, the assembled reads ranged between 500 to 1696 bp and averaged 869 bp in length. This highlighted that our sequencing and gene assembly workflow was unable to consistently assemble the reads into the full-length sequences, particularly when considering studies that were able to generate 1430 bp 16S rRNA gene sequences of elephant gut bacteria using a Nanopore sequencer^31^, or those performing similar gene reconstruction techniques with alternative assemblers^32^. For the assembly step in our workflow, we implemented the MATAM software^33^. This tool has a documented tendency to overpredict and generate a number of short, fragmented scaffolds compared to other assemblers, which may serve to explain a portion of the variability in read lengths^34^. Short-read assembly algorithms also often struggle to differentiate between homologous sequences derived from conserved regions of the 16S rRNA gene, contributing to the generation of shorter assemblies^34^. The use of databases containing partial sequences of the target gene may also contribute to the generation of near full-length 16S rRNA amplicon assemblies. It is worth noting that the SILVA reference database primarily consists of high-quality near-full-length sequences^35^, as opposed to complete 16S rRNA gene sequences, which may account for some of the discrepancy in read lengths. However, despite this variation and reduced mean read length, assemblies achieved the expected concordance with the theoretical composition of the microbial community standard.

Although the association between the composition of the theoretical and sequenced mock communities was considered very weak, the discrepancies contributing to this result are likely to have arisen due to technical factors unrelated to the agreement in bacterial species identity, as seven of the eight expected species were present. Deviations in abundance from the theoretical composition may have occurred as a result of the DNA extraction method, as is likely the case with the absent gram-positive *Limosilactobacillus fermentum* species, or the PCR primer choice, which may contribute to a biased representation of various bacterial clades^32,36^. Interestingly, the abundances of *Staphylococcus aureus*, *Salmonella enterica*, *Listeria monocytogenes,* and *Bacillus subtilis* in the sequenced mock sample were over-represented in similar proportions to recent work contrasting the theoretical composition of the ZymoBIOMICS microbial standard with near-entire *rrn*^37^. This highlights the potential variability in the commercial product relative to stipulated proportions^37^, in addition to variation introduced through laboratory and bioinformatic processing. However, relative abundance is compositional in nature, meaning that the similarity of our sequenced microbial standard and the abundances of the aforementioned taxa in the nanopore-sequenced mock communities of Petrone et al. (2023), may have been coincidental and was likely caused by the over- or under-representation of the other bacterial species^37^. Taxonomic classification with the SILVA 138.1 reference database did not appear to misidentify any of the bacterial taxa present in the microbial standard, improving confidence in the species-level assignments.

The alpha diversity indices revealed differences in intra-sample diversity between the sample type and captivity status groups. BALF samples exhibited higher intra-sample diversity compared to the ETT and TW samples. Free-ranging elephant samples had alpha diversity estimates that suggested greater bacterial diversity exists in the respiratory communities of elephants in their natural environment in contrast to those in captivity. Similar reductions in bacterial diversity have been previously identified in the gut microbiome of managed (zoo) African elephants compared to their free-ranging counterparts, further highlighting the impact of the environment on microbial diversity within these animals^38^. Multidimensional Scaling (MDS) analysis of the respiratory bacterial profiles of the elephants revealed a statistical trend (*p* = 0.085, PERMANOVA) toward sample type divergence, based on the deviation of inter-sample diversity of the TW and ETT samples (*p* = 0.029, *p*_*adj*_ = 0.087). Given that ETT samples were collected by rinsing the endotracheal tube with sterile saline following the collection of BALF from the lower respiratory tract of the elephant, a high degree of similarity in the bacterial communities of these sample types is expected. However, the segregation of the ETT from TW samples, although not statistically significant, suggests the occurrence of distinct bacterial signatures characteristic of the two respiratory tract compartments i.e., the lower respiratory tract and the trunk.

Through the application of the short-read 16S rRNA amplicon sequencing technique and gene assembly workflow to opportunistically collected samples, novel insights into the composition of the respiratory microbiota of captive (zoo) and free-ranging elephants could be described. The respiratory samples were dominated by Proteobacteria, Actinobacteriota, Firmicutes, and Bacteroidota phyla. Whilst the abundance of these phyla differs across animal species, these findings are in agreement with a recent meta-analysis of 16S rRNA gene datasets characterising the respiratory microbiota of six different domestic animals^21^. The presence of similar bacterial phyla across mammals may arise from common respiratory patterns, which could be attributed to the relatively uniform anatomy and physiology of the respiratory tracts among different mammalian species^39^. Results from the meta-analysis serve to further validate our genus-level findings, through their identification of similar common bacterial genera, particularly *Streptococcus*, *Pseudomonas*, *Moraxella,* and *Acinetobacter*, amongst others, in respiratory profiles of a wide range of animals^21^.

Interesting observations emerged from the visual assessment of prominent bacterial genera and species within the samples. *Rothia, Enhydrobacter,* and *Moraxella* species exhibited concurrent presence in five of the eight TW samples, suggesting species-specific involvement of *Rothia amarae*, *Enhydrobacter aerosaccus*, *Moraxella osloensis*, and *Dermabacter hominis* in this sample type. *Rothia amarae* species have been frequently isolated from environmental sources^40^. Furthermore, animal-associated *Rothia* species are prevalent members of the upper respiratory tract with documented abilities of exopolysaccharide synthesis for biofilm formation, complex carbohydrate metabolism, and antimicrobial compound production^40^. *Enhydrobacter aerosaccus* was first characterised following isolation from eutrophic lake water, but has been more recently implicated in various niches, including host-associated microbiomes (i.e. skin and blood)^41^. *Moraxella* species, including *Moraxella osloensis*, are recognised as common bacterial inhabitants of mucosal surfaces and have been isolated from the respiratory tracts of a range of mammalian hosts^42^ and from multiple environmental sources^43^. Thus, the predominance of *Rothia amarae*, *Enhydrobacter aerosaccus* and *Moraxella osloensis* in the elephants’ trunks is unsurprising, as the trunk represents a unique interface between the animal’s upper respiratory tract and its environment, given its function in breathing, feeding, olfactory perception and tactile exploration^44^.

*Pseudomonas* was notably present and largely dominant in seven of the ten BALF samples collected from free-ranging elephants and may, therefore, represent a bacterial genus characteristic of this sample type. Of the members of the genus dominating the BALF profiles, *Pseudomonas kribbensis* and *Pseudomonas koreensis* have been previously isolated from garden^45^ and agricultural soils^46^ and *Pseudomonas aeruginosa* has been frequently implicated in respiratory disease^46^. The identification of these *Pseudomonas* species in elephants is not unexpected, given their direct and regular exposure to the ground, soil and dust. Furthermore, the lower respiratory tracts of elephants are not sterile as organic matter is often found deep in the airways during endoscopies (M.A. Miller, personal communication, 12 November 2023). Therefore, this finding highlights the possible novel contributions of less common environmental *Pseudomonas* species, potentially introduced through their interaction with the natural environment, in the lung microenvironment of elephants. On the other hand, the predominance of *Pseudomonas aeruginosa* in some of the profiles may suggest respiratory infection or compromised lung function in some of these animals, considering the bacterium’s implication in respiratory disease. *Pseudomonas aeruginosa* is an opportunistic pathogen in both humans and animals and is often involved in persistent biofilm infections^47^. Studies have demonstrated that *Pseudomonas aeruginosa* induces a robust inflammatory response which, in the context of trained immunity, may play a beneficial role in modulating innate and adaptive immunity, enhancing the host’s response towards other microorganisms, including mycobacteria.

Several limitations exist within the current study. In addition to the aforementioned biases introduced by PCR primers and the reference databases used for taxonomic classification, bacterial community structure represented using targeted metagenomic sequencing methods are subject to further inaccuracies. However, this issue is not unique to the current study, as it affects most microbiome studies that target the 16S rRNA gene. Other common limitations include confounding factors that influence the presence and abundance of microbiota. For instance, in the current study, variables including, but not limited to, the sampling season, age, sex, and infection status of the elephants may have influenced the respiratory microbial composition. However, sample collection could not be planned, and was instead performed opportunistically when elephants were immobilised for routine management procedures. Moreover, whilst veterinarian-assisted TW samples could be collected from free-ranging elephants during immobilisation, captive elephants were not trained to provide TW samples and thus, were not collected and included in this study. This has led to limited sample size and uneven sample type distribution, which has hindered the ability to conduct statistically sound analyses. Sample type and captivity status also present as confounding variables of each other which may limit the ability to make any definitive conclusions. Another limitation pertains to the transportation and storage of the samples at − 20 °C. The gold standard is flash-freezing or storage at – 80 °C^48^; however, this presents a challenge when collecting samples in remote areas, given the limited access to the required equipment. All samples were, therefore, consistently stored in the available − 20 °C equipment and facilities. While we acknowledge the limitations hereof, we anticipate that these storage conditions have not significantly biased bacterial abundances, considering the results of a recent study that showed that unpreserved saliva samples stored at − 15 °C maintained their microbial composition and integrity^49^.

Despite these challenges, this exploratory study has provided the first glimpse into the respiratory bacterial communities harboured within these animals at species level using a short-read full-length 16S rRNA amplicon sequencing technique and assembly workflow. Future studies should consider refining the sequencing technique and microbiome-related analysis workflow. Selection of better suited primer sets, use of mock communities with representation of bacterial clades characteristic of the microbial niche of interest^48^, curated reference databases for taxonomic classification, and well-maintained 16S rRNA amplicon assembly programmes may contribute to improving species-level taxonomic assignments and characterisation of bacterial communities. In the context of the elephant respiratory microbiota, a larger sample size may allow for the identification of novel microbial species and a greater understanding of the significance of the respiratory microbiota’s complexity in the context of elephant health, infection response, and the environment with which it interacts.

## Conclusion

The results presented in the current study demonstrate the viability of the adapted sequencing technique for high-quality partial to near full-length short-read 16S rRNA gene sequencing on the Illumina iSeq 100 platform. Application of the technique to mock microbial community standards and African elephant respiratory samples enabled accurate taxonomic classification of bacteria at genus and species level. It is essential to exercise caution when interpreting these findings, given the limited sample size and the multitude of potentially influential external factors. Further investigation into the respiratory microbiota of free-ranging and captive elephants is warranted to validate the tentative visually-identified bacterial patterns.

## Methods

### Specimen collection

A total of 17 African elephants were sampled, four of which were captive zoo elephants and 13 of which were free-ranging individuals (Supplementary Fig. S5). From these, 12 BALF samples, three ETT samples and eight TW samples were opportunistically collected from immobilized elephants during routine management procedures using previously reported methods^50–52^. The samples described were not primarily collected for microbiome analysis, but rather acquired as part of an approved project to evaluate respiratory samples using mycobacterial culture. Approximately 150 mL of each respiratory sample was collected in a 500 mL sterile suction vacuum container. Samples were transported to the laboratory on cooled ice bricks in single shipments at − 20 °C within four hours of collection. Upon receipt, the total volume was divided into three separate Corning™ Falcon™ 50 mL Conical Centrifuge Tubes (ThermoFisher Scientific, Waltham, MA, USA) per animal. The respiratory samples were concentrated through centrifugation at 2000×*g* for 30 min and decanting of supernatant in excess of 4 mL, followed by storage at − 20 °C until extraction.

This study received ethical approval from the Stellenbosch University Animal Care and Use Committee, as indicated by reference numbers SU-ACU-2018–6308 and SU-ACU-2021-21572. Permission to conduct animal research under the provisions of Section 20 of the Animal Diseases Act was granted by the South African Department of Agriculture, Land Reform and Rural Development (DALRRD), South Africa, under permit numbers 12/11/1/7/2 and 12/11/1/7/6E (JD). A Threatened or Protected Species (TOPS) permit was obtained through the Department of Environmental Affairs (DEA Standing Permit S02556; S65805 and DEA Registration Certificate 29416; 02256). All animal handling and sampling procedures were carried out by South African Veterinary Council (SAVC) registered veterinarians in accordance with established standard operating procedures.

### Microbial DNA extraction

Microbial DNA was extracted from samples using the QIAGEN DNeasy^®^ Blood and Tissue kit (Qiagen, Hilden, Germany) following established protocols^53^. A suspension of 75 μL ZymoBIOMICS™ Microbial Community Standard (Zymo Research, Irvine, CA) and 175 μL of UltraPure DNase/RNase-Free Distilled Water (ThermoFisher Scientific) was extracted, enabling analysis of this technique against a theoretical composition for comparative purposes. Extracted DNA was quantified with the Qubit dsDNA High Sensitivity Assay kit on the Qubit 4 fluorometer (ThermoFisher Scientific) according to the manufacturer’s instructions.

### 16S rRNA sequencing

A brief overview of the library preparation, sequencing and bioinformatic analysis is presented in Fig. 1a. The full-length 16S rRNA gene was amplified from extracted microbial DNA using a universal 27F and 1492R primer set^24^ ligated to 5′ Illumina Nextera kit-specific overhangs^54^. Polymerase chain reactions (PCRs) were performed in 50 μL reaction volumes of 10 μL 5× Takara PrimeSTAR GXL buffer (Takara Bio, Shiga, Japan), 4 μL of dNTP mixture (Takara Bio), 1.5 μL of 5 μM 27F and 1492R primers, 1 μL of Takara PrimeSTAR GXL DNA Polymerase (Takara Bio), 5 ng of DNA template, and PCR-grade water up to the total volume. The PCR conditions included 30 cycles of denaturation at 98 °C for 10 s, annealing at 60 °C for 15 s, and extension at 68 °C for 1 min and 30 s. Amplicon sizes were verified on a 1% (w/v) agarose gel, prior to post-amplification purification using the NucleoMag PCR kit (Macherey–Nagel, Düren, Germany), according to the manufacturer's instructions. The concentration of the clean PCR products for each sample was evaluated using the Qubit dsDNA Broad Range assay on the Qubit 4 fluorometer (ThermoFisher Scientific) and normalised to 100 ng/μL in a 30 μL volume, following confirmation of amplicon purification on a 2% (w/v) agarose gel.

The protocol for sequencing hypervariable regions of the 16S rRNA gene on Illumina instruments typically has an indexing PCR step following PCR clean-up that attaches dual indices and sequencing adaptors to purified amplicons^54^. However, amplicon libraries were prepared using the Illumina Nextera XT DNA Flex Library Prep kit (Illumina, San Diego, CA), which included additional tagmentation and clean-up steps, and were performed according to the manufacturer’s instructions. Libraries were cleaned and the average amplicon size (483.46 bp) was confirmed with the Tape Station 4200 (Agilent, Santa Clara, CA). Since approximately 120–150 bp of the visualised library size could be attributed to sequencing adapters and barcodes, this means that in some cases, a gap between the reads may have existed, but for the most part the entire insert was sequenced.

Pooled libraries and a 10% (v/v) spike-in of PhiX Control V3 (Illumina), both diluted to 50 pM, were loaded onto the Illumina™ iSeq 100 i1 V2 cartridge and sequenced in 2 × 150 bp reads on the Illumina™ iSeq 100 instrument. Samples were demultiplexed using the GenerateFASTQ module on the Illumina™ iSeq 100 instrument following sequencing. Run quality and performance metrics were viewed on Illumina™ Sequence Analysis Viewer version 2.4.7.

### Processing raw reads

The R1 and R2 fastq-formatted sequence files for each sample were downloaded onto a local machine from Illumina BaseSpace Sequence Hub and analysed using 16S-amplicon-seq^55^, a custom Nextflow workflow (https://github.com/laurencmartin/16S-amplicon-seq) developed for paired-end read concatenation, 16S rRNA gene assembly, and taxonomy and amplicon sequence variant (ASV) table generation for input into the *phyloseq*^56^ (version 1.42.0) package in RStudio (R Foundation for Statistical Computing)^57^, alongside sample-specific metadata (Fig. 1b). Briefly, following input into the 16S-amplicon-seq workflow, raw fastq files were directed to *FastQC*^58^ (version 0.11.9) to generate summary reports detailing basic read and sequence-related statistics (i.e., read count, sequence length, percentage GC content, quality scores and adapter content) for each sample. Reads were trimmed using *Trim Galore*^59^ (version 0.6.5) with the following parameters: *–q 30*, *–length 50*, *–clip_R1* and *–clip_R2* flags, to retain bases with quality scores ≥ 30 and reads with a minimum length of 50 bases, and ensure 5′ region of the sequences were removed to account for reduced read quality. Forward and reverse reads were concatenated to ensure compliance with the *MATAM*^33^ (version 1.6.0) gene assembly algorithm’s input requirements. The *MATAM* assembly module was executed using default parameters.

Sequence abundance estimates, required for ASV table generation, were extracted from MATAM’s output. Taxonomic classification was performed on the resultant assembly files using the *SILVA* 138.1 prokaryotic SSU taxonomic training dataset formatted for *DADA2* (silva_nr99_v138.1_wSpecies_train_set.fa.gz)^35^ by implementing the RDP Naïve Bayesian Classifier algorithm^60^. Taxonomy tables and abundance files were integrated into sample-specific amplicon sequence variant (ASV) tables. Lastly, each newly generated ASV and taxonomy table was merged to create a single *phyloseq*-compatible ASV and taxonomy comma separated value (csv) file for the processed samples.

The *phyloseq*^56^ *tax_glom* function is unable to agglomerate taxa with identical names at lower taxonomic levels with differing taxonomy at higher ranks, which impacts its ability to collapse bacterial taxa to species-level rank. To account for this, taxonomy tables were redirected into an automated taxonomy renaming tool (https://github.com/laurencmartin/taxa-table-tool) that denominates the input to ensure unique species labels for all genera that share the same species names^55^. A denominated taxonomy table was then re-imported into R (version 4.3.1) for diversity analyses using scripts contained in the Rhea^61^ pipeline and regeneration of an updated *phyloseq* object for subsequent agglomeration of the taxa at genus and species level and relative abundance visualisation.

### Mock microbial community comparison

The comparison of the mock community profile to the theoretical composition of the ZymoBIOMICS™ Microbial Community Standard was performed using the *checkZymoBiomics* function of the *chkmocks* package^36^ in R. The *checkZymoBiomics* function uses FASTA files containing full-length 16S rRNA gene sequences of the expected microbes in the commercial standard for the taxonomic classification of the ASVs present in the mock community of interest. It then assesses the degree of similarity between the relative taxon abundances of the theoretical composition of the standard reported by the supplier, derived using the formula: (total genomic DNA (g) × unit conversion constant (bp/g)/genome size (bp)) × 16S/18S copy number per genome), against the composition of mock community with a Spearman’s rank correlation test^36^. The sequenced mock and theoretical composition were visualised as a stacked bar plot using the *plotZymoDefault* function of the same package.

### Diversity analyses

Bacterial composition and diversity analyses at phylum, genus, and species level were conducted in R. The ASV table was normalised using Total Sum Scaling (TSS) procedures, as scripted for in the Rhea pipeline, whereby each sample’s counts were divided by their sample size and subsequently scaled up by the smallest sample’s size across the dataset^61^. This scaling method was implemented to minimise data loss or the introduction of variance to the data, which commonly occurs when rarefying (randomly sub-sampling reads to a specific sequencing depth)^61^. Rarefaction curves were generated, using the same script, to assess whether the sequencing depth was adequate to capture the diversity within the samples. An abundance filtering cut-off of 0.10% was applied to the dataset to prevent downstream analysis of spurious reads^61^. Alpha and beta diversity analyses were performed with scripts supplied in the Rhea pipeline^61^. Shannon, effective Shannon, Simpson and effective Simpson alpha diversity indices were used to assess intra-sample variation and Multidimensional (MDS) plots of generalized UniFrac distances were used to visualise the distribution of the samples with reference to their phylogenetic relatedness to evaluate beta diversity. Assessment of differences in beta diversity centroids and dispersion among the studied groups was performed using a Permutational Multivariate Analysis of Variance (PERMANOVA) by employing the *adonis2* function nested within the *vegan*^62^ package. Significance was defined as *p* < 0.05.

### Taxon abundances

Following generation of a phyloseq object from the normalised ASV table, the denominated taxa table and metadata file, normalised ASV counts were transformed into proportions and visualisation of the top 25 abundant bacterial genera and species across samples using facet-wrapped stacked bar charts was performed. The proportional average abundances and standard deviations of the top phyla, genera, and species across groups were obtained using the *get_abundances* function from the *microbiomeutilities*^63^ package (version 1.00.17).

### Supplementary Information

Supplementary Figures.

Supplementary Information.

## Data Availability

The targeted V1–V9 metagenomic sequencing data generated in this study has been deposited under NCBI Project ID PRJNA1102949. The 16S-amplicon-seq workflow is available at https://github.com/laurencmartin/16S-amplicon-seq.
