# Supplementary material for: Short-read full-length 16S rRNA amplicon sequencing for characterisation of the respiratory bacteriome of captive and free-ranging African elephants (Loxodonta africana)
Source: Sci Rep. 2024 Jun 26;14:14768. doi: 10.1038/s41598-024-65841-4 (PMC11208578; doi:10.1038/s41598-024-65841-4)
Supplement: Supplementary file 1 — Supplementary Figures. [file 41598_2024_65841_MOESM1_ESM.docx]

Short-read full-length 16S rRNA amplicon sequencing for characterisation of the respiratory bacteriome of captive and free-ranging African elephants (*Loxodonta africana*)

Lauren C. Martin^1,2,3^, Michaela A. O’Hare^2,3^, Giovanni Ghielmetti^4,5^, David Twesigomwe^6,7^, Tanya J. Kerr^4^, Rachiel Gumbo^4^, Peter E. Buss^8^, Natasha Kitchin^1,2^, Sian M.J. Hemmings^1,2^ , Michele A. Miller^4, #^, Wynand J. Goosen^4, #, *^

^1^ Department of Psychiatry, Faculty of Medicine and Health Sciences, Stellenbosch University, PO Box 241, Cape Town 8000, South Africa

^2^ South African Medical Research Council/Stellenbosch University Genomics of Brain Disorders Unit, Cape Town, South Africa

^3^ Division of Molecular Biology and Human Genetics, Faculty of Medicine and Health Sciences, Stellenbosch University, PO Box 241, Cape Town 8000, South Africa.

^4^ South African Medical Research Council Centre for Tuberculosis Research, Division of Molecular Biology and Human Genetics, Faculty of Medicine and Health Sciences, Stellenbosch University, PO Box 241, Cape Town 8000, South Africa.

^5^ Section of Veterinary Bacteriology, Institute for Food Safety and Hygiene, Vetsuisse Faculty, University of Zurich, Winterthurerstrasse 270, 8057 Zurich, Switzerland

^6^ Sydney Brenner Institute for Molecular Bioscience, Faculty of Health Sciences, University of the Witwatersrand, Johannesburg, South Africa

^7^ Division of Human Genetics, National Health Laboratory Service, and School of Pathology, Faculty of Health Sciences, University of the Witwatersrand, Johannesburg, South Africa

^8^ South African National Parks, Veterinary Wildlife Services, Kruger National Park, Skukuza, South Africa

Martin ([lcmartin@sun.ac.za](mailto:lcmartin@sun.ac.za)); O’Hare ([21772207@sun.ac.za](mailto:21772207@sun.ac.za)); Ghielmetti ([gghielmetti@sun.ac.za](mailto:gghielmetti@sun.ac.za)); Twesigomwe ([david.twesigomwe@wits.ac.za](mailto:david.twesigomwe@wits.ac.za)); Kerr ([tjkerr@sun.ac.za](mailto:tjkerr@sun.ac.za)); Gumbo ([rachy@sun.ac.za](mailto:rachy@sun.ac.za)); Buss ([peter.buss@sanparks.org](mailto:peter.buss@sanparks.org)); Kitchin ([natashak@sun.ac.za](mailto:natashak@sun.ac.za)); Hemmings ([smjh@sun.ac.za](mailto:smjh@sun.ac.za)); Miller ([miller@sun.ac.za](mailto:miller@sun.ac.za)); Goosen ([wjgoosen@sun.ac.za](mailto:wjgoosen@sun.ac.za))

^#^ These authors share senior authorship

Corresponding author: Dr Wynand J. Goosen*

Correspondence address: South African Medical Research Council Centre for Tuberculosis Research, Division of Molecular Biology and Human Genetics, Faculty of Medicine and Health Sciences, Stellenbosch University, PO Box 241, Cape Town 8000, South Africa

Tel: +27 73 885 3266; E-mail: [wjgoosen@sun.ac.za](mailto:wjgoosen@sun.ac.za)

**Supplementary Material**


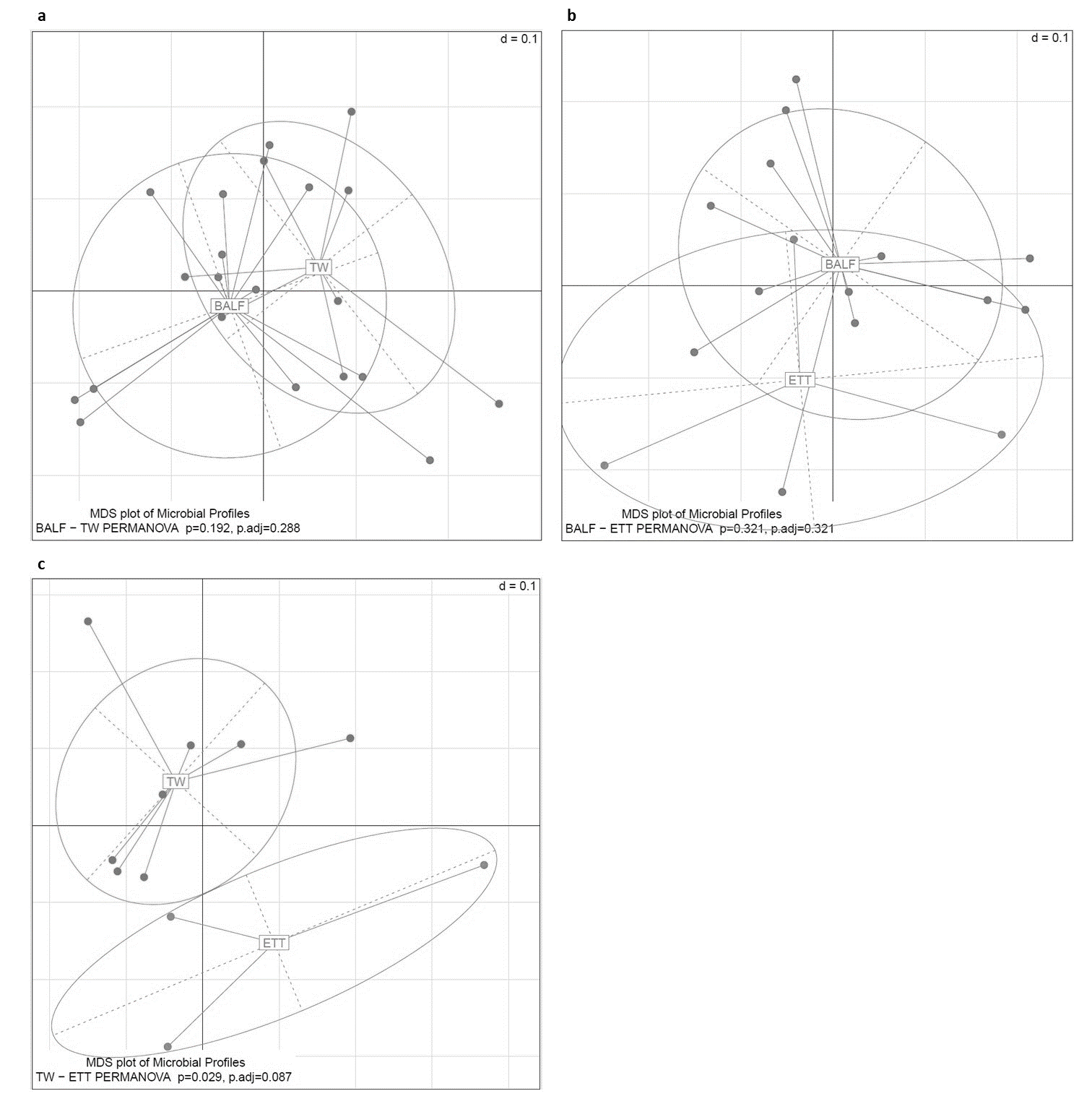


**Supplementary Fig. S1: Pairwise Multidimensional Scaling (MDS) plots of generalized Unifrac dissimilarities derived from the bacterial communities of BALF, ETT and TW respiratory samples of captive (zoo) and free‑ranging elephants.** Pairwise comparisons between (**a**) BALF and TW, (**b**) BALF and ETT, and (**c**) TW and ETT samples are displayed. Pairwise comparison revealed near significant differences in the intersample diversity of the ETT and TW samples (*p* = 0.029, *p*_adj_ = 0.087).


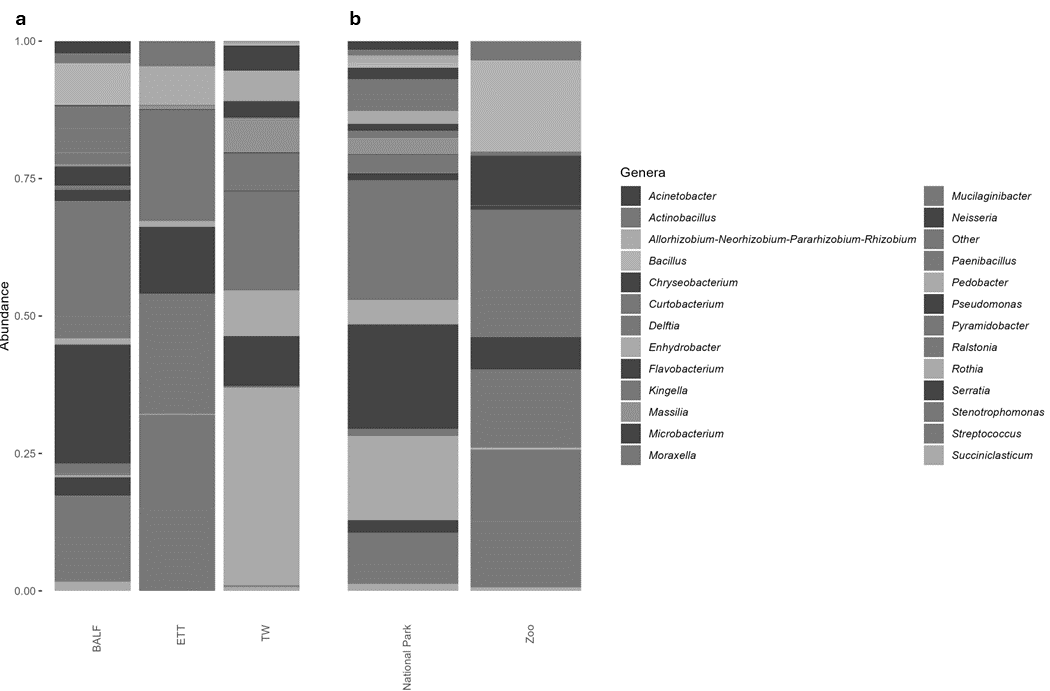


**Supplementary Fig. S2: Averaged abundances of the 25 most abundant bacterial genera present in the elephant respiratory samples.** Stacked bar charts are facetted by (**a**) sample type and (**b**) captivity status. Abbreviations: BALF, bronchoalveolar lavage fluid; TW, trunk wash; ETT, endotracheal tube wash.


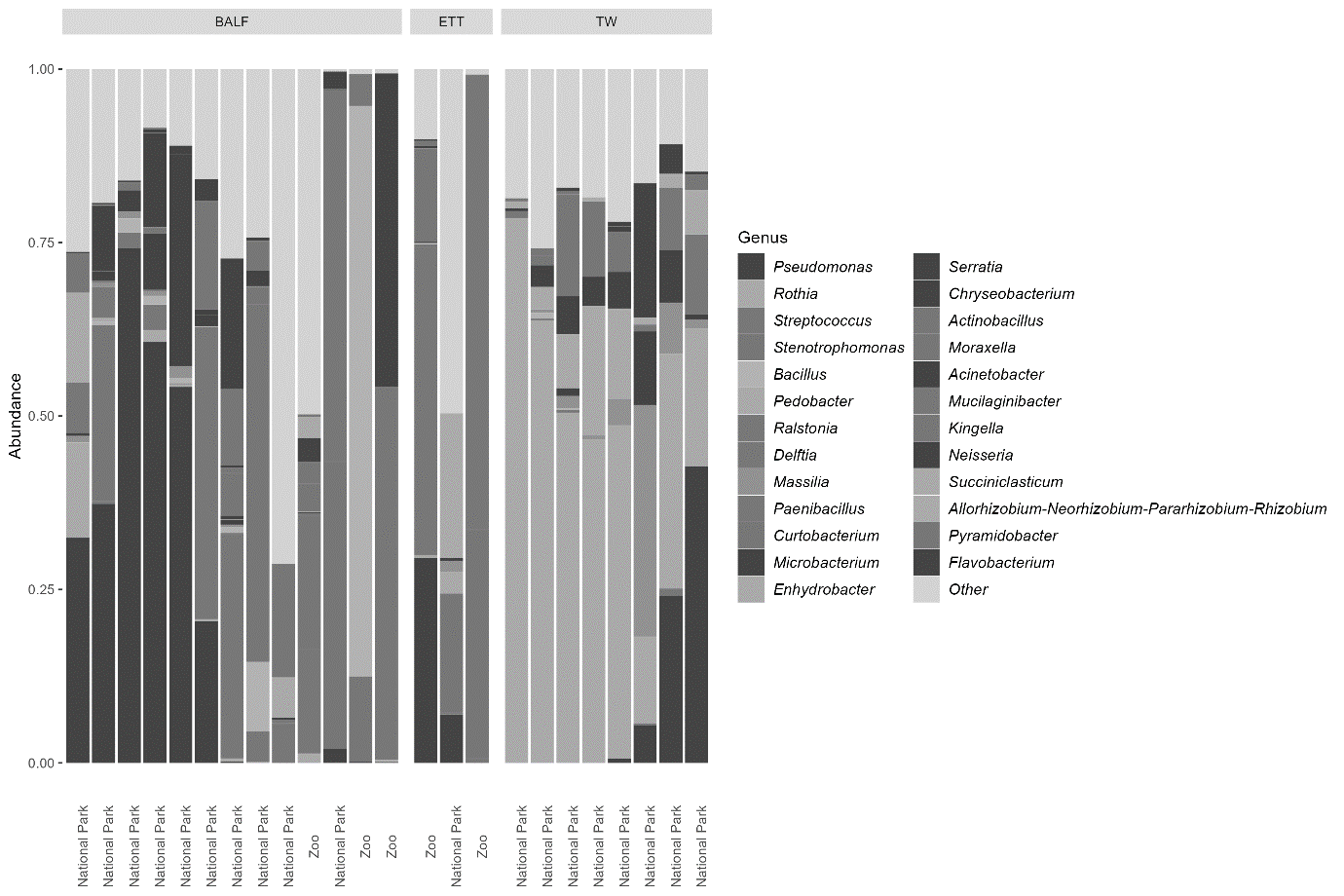


**Supplementary Fig. S3: Top 25 most abundant bacterial genera present in BALF, ETT and TW samples collected from captive (zoo) and free-ranging elephants.** Relative abundances of these genera are displayed with stacked bar charts stratified by sample type. Samples are ordered according to Bray‑Curtis dissimilarity. Abbreviations: BALF, bronchoalveolar lavage fluid; TW, trunk wash; ETT, endotracheal tube wash.


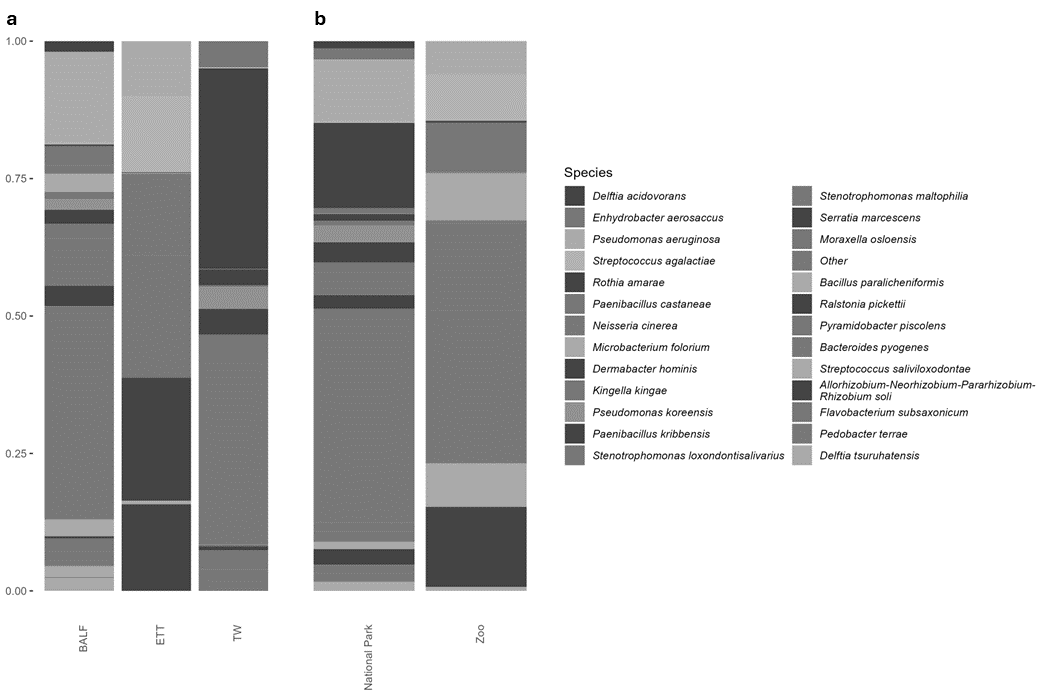


**Supplementary Fig. S4: Averaged abundances of the 25 most abundant bacterial species present in the elephant respiratory samples.** Stacked bar charts are facetted by (**a**) sample type and (**b**) captivity status. Abbreviations: BALF, bronchoalveolar lavage fluid; TW, trunk wash; ETT, endotracheal tube wash.

**Supplementary Fig. S5: Elephant respiratory samples available for microbiome analysis.** Abbreviations: BALF, bronchoalveolar lavage fluid; TW, trunk wash; ETT, endotracheal tube wash.
